# Mycotoxin Cocktail in the Samples of Oilseed Cake from Early Maturing Cotton Varieties Associated with Cattle Feeding Problems

**DOI:** 10.3390/toxins7062188

**Published:** 2015-06-12

**Authors:** Agha W. Yunus, Michael Sulyok, Josef Böhm

**Affiliations:** 1 Animal Nutrition Program, Animal Sciences Institute, National Agricultural Research Centre, Park Road, Islamabad 45500, Pakistan; 2 Center for Analytical Chemistry, Department IFA-Tulln, University of Natural Resources and Life Sciences Vienna (BOKU), Konrad Lorenzstrasse 20, Tulln A-3430, Austria; E-Mail: michael.sulyok@boku.ac.at; 3 Institute of Animal Nutrition and Functional Plant Compounds, University of Veterinary Medicine Vienna, Veterinärplatz 1, Vienna 1210, Austria; E-Mail: josef.boehm@vetmeduni.ac.at

**Keywords:** cattle, cottonseed, toxicity, mycotoxin

## Abstract

Cottonseed cake in South East Asia has been associated with health issues in ruminants in the recent years. The present study was carried out to investigate the health issues associated with cottonseed cake feeding in dairy animals in Pakistan. All the cake samples were confirmed to be from early maturing cotton varieties (maturing prior to or during Monsoon). A survey of the resource persons indicated that the feeding problems with cottonseed cake appeared after 4–5 months of post-production storage. All the cake samples had heavy bacterial counts, and contaminated with over a dozen different fungal genera. Screening for toxins revealed co-contamination with toxic levels of nearly a dozen mycotoxins including aflatoxin B_1_ + B_2_ (556 to 5574 ppb), ochratoxin A + B (47 to 2335 ppb), cyclopiazonic acid (1090 to 6706 ppb), equisetin (2226 to 12672 ppb), rubrofusarin (81 to 1125), tenuazonic acid (549 to 9882 ppb), 3-nitropropionic acid (111 to 1032 ppb), and citrinin (29 to 359 ppb). Two buffalo calves in a diagnostic feed trial also showed signs of complex toxicity. These results indicate that inappropriate processing and storage of the cake, in the typical conditions of the subcontinent, could be the main contributory factors regarding the low quality of cottonseed cake.

## 1. Introduction

Cotton is a major cash crop in Southeast Asia as over 45% of the total area under oilseed crops in the region is dedicated to cotton cultivation. In Pakistan, the production of cottonseed was estimated to be 2.2 million tons in the year 2013–2014 [1]. This means that approximately 0.64 million tons of cottonseed cake (CSC) was available for livestock feeding in the country. Cottonseed cake (CSC) is the byproduct of mechanical oil extraction from cottonseed, and it is the most commonly used vegetable protein source for large ruminants in the Indian subcontinent.

In recent years, the quality of CSC has been a cause of public concern as many of its batches were reported to cause health issues in cattle/buffaloes. In some instances, CSC was alleged to result in a mysterious syndrome and mortality in large ruminants [2,3]. The introduction of GM cotton varieties in the year 2002 is commonly accused, without any investigations, to be the cause of such cases. The present investigations were therefore aimed at studying the CSC samples suspected to cause toxicity in cattle and buffaloes. A short survey of the resource persons and complainant farmers was conducted before any laboratory analyses.

## 2. Results and Discussion

Out of the six CSC samples included in the study, two (CSC-Okara, CSC-ICT-1) were associated with cattle mortality (16% milking animals). No postmortem findings were reported for the sample CSC-Okara, while the authors noted pale livers upon post mortem of the cattle fed the CSC-ICT-1. Unfortunately, these 2 samples were not available in quantities sufficient for any diagnostic feeding test. Three samples of CSC were found to result in reduced milk production, respiratory distress, and diarrhea followed by feed refusal. These symptoms of toxicity could be reversed after withdrawal of the CSC.

The information collected from the stakeholders indicated that the freshly produced CSC is not implicated in buffalo/cattle poisoning. In opinion of the oil expellers, the complaints with CSC feeding arise after several months of post-production storage, indicating a possibility of fungal invasion during hot and humid climatic conditions. Additionally, past incidences of monsoon floods (August/September 2011 and 2012) in the Indus valley (Sindh), among the main cotton growing areas, were considered as the main contributory factor by the retailers of CSC.

### 2.1. Cry Proteins, Macronutrient, and Heavy Metal Analyses

The levels of Cry1Ac protein in the samples were 1.2 ± 0.5 μg/g of fresh weight, showing that all the samples were from early maturing transgenic cotton varieties. No significant differences among different batches were found regarding nutrient composition or fiber quality (Supplementary Table S1), excluding adulteration as a possible cause of low quality of the CSC samples. The cadmium content exceeded the maximum permitted level of 1.0 mg/kg of feed in almost all the CSC samples associated with feeding problems (Supplementary Table S2). However, the noted levels of heavy metals were not enough to result in toxicity. It has been reported that the levels of cadmium in soil from different ecological zones of Pakistan are in safe ranges and lower than the world’s average [4]. With this factor in view, it is reasonable to expect that the source of cadmium in the problematic samples was the inappropriate metal used in the oil pressing industry. The contamination of the samples with cadmium appeared to be independent of the origin (north *vs.* south Indus river, *i.e.*, Sindh *vs.* Punjab provinces).

**Table 1:** Mycotoxins (μg/kg) noted in high quantities in the cottonseed cake samples.

Mycotoxins	Fungal genera. suspected	Samples with mortality		Samples with feed refusal			Control
		CSC	CSC	CSC	CSC	CSC	
		Okara	ICT-1	ICT-2	ICT-3	ICT-4	
Aflatoxin B_1_	*Aspergillus*	5147	1771	1448	1212	517	661
Aflatoxin B_2_	*Aspergillus*	427	136	98	99	39	48
Aflatrem ^†^	*Aspergillus*	12,730	n.d.	8798	14,670	10,490	3970
Cyclopiazonic acid	*Aspergillus/Penicillium*	6706	2029	1819	2258	1090	736
Ochratoxin A	*Aspergillus/Penicillium*	1629	816	375	1248	898	36
Ochratoxin B	*Aspergillus/Penicillium*	706	399	103	399	288	11
Citrinin	*Aspergillus/Penicillium*	153	359	95	192	97	29
Andrastin A ^†^	*Aspergillus/Penicillium*	5644	n.d.	2239	10,340	12,030	14,200
Paspalitrem A ^†^	*Aspergillus/Claviceps*	89,170	11,870	15,150	107,600	19,680	17,550
Paspalin ^†^	*Aspergillus/Claviceps*	18,520	6484	3771	34,400	4982	5975
Paspalinine ^†^	*Aspergillus/Claviceps*	3687	693	1598	5290	3131	2766
Equisetin	*Fusarium*	2226	9818	12,672	3840	5175	3573
Rubrofusarin	*Fusarium*	211	1125	644	81	449	6392
Tenuazonic acid	*Alternaria/Phoma*	2917	9882	4382	1542	2237	549
3-Nitropropionic acid	*Aspergillus/Arthrinium*	337	759	1032	111	185	300

n.d. = less than detection limit, CSC = cottonseed cake, ICT = Islamabad capital territory, FSD = Faisalabad (Punjab province), SDT = Shahdadkot (Sindh province); Control = cottonseed cake collected from a state-owned cattle feed mill in Islamabad Capital Territory. ^†^ Number denotes peak area (standard not available).

### 2.2. Fungal and Bacterial Toxins

Results from the microbial metabolite screening are presented in Table 1 and Table 2. In total, 72 fungal metabolites were detected in these samples, indicating heavy invasion of toxigenic *Aspergillus*, *Penicillium*, *Fusarium*, *Alternaria*, *Arthrinium*, and *Curvularia* spp. besides others. The *Aspergillu*s (*flavus*), *Fusarium*, and *Alternaria* spp. could also be isolated from the samples. Aflatoxins and ochratoxins were present in all the tested samples. However, exceptionally high quantities of the carcinogenic mycotoxins aflatoxins (total B_1_ + B_2_ = 5575 μg/kg) and ochratoxins (A + B = 2335 μg/kg) were found in the CSC-Okara, which was suspected to cause mortality in a mixed herd of buffalo and cattle. The other sample suspected to cause mortality (ICT-1) was the second highest in the total aflatoxins level, *i.e.*, 1907 μg/kg, while the third highest regarding the level of ochratoxins, *i.e.*, 1215 μg/kg. The levels of tremorgenic mycotoxins, *i.e.*, andrastin A, aflatrem, paspalitrem A, paspalin, and paspalinine in the latter sample (CSC-ICT-1) were lower compared to the other samples. The postmortem findings of eight cows died after consuming this sample revealed pale livers, which implies that the main toxic effects in this case could be of aflatoxins. The symptoms and postmortem findings associated with the CSC-Okara sample were not revealed to us. In the absence of such information it is impractical to infer which mycotoxin was primarily responsible for mortality at that farm. However, the exceptionally high levels of aflatoxins and ochratoxins in the CSC-Okara sample indicate that these might also be the main causes of the mortality observed with feeding of this sample.

**Table 2:** Fungal metabolites noted in low concentrations (μg/kg) in the cottonseed cake samples.

Metabolite	Control (μg/kg)	Other CSC samples			Metabolite	Control (μg/kg)	Other CSC samples		
		*n* (+ive)	Median (μg/kg)	Max (μg/kg)			*n* (+ive)	Median (μg/kg)	Max (μg/kg)
*Aspergillus/Penicillium:*					*Fusarium spp.:*				
Averufin	67.8	5	137.1	332.1	Monoactoxyscirpenol	2.1	5	4.3	35.8
Averufanin *	4.5	5	16.3	39.2	Moniliformin	7.1	5	1.8	8.1
Nidurufin *	1.1	5	3.1	5.4	Enniatin B	n.d.	5	0.2	0.4
Averantin	2.3	5	6.3	24.7	Enniatin B_1_	n.d.	3	0.1	0.4
Norsolorinic acid *	1.5	5	4.0	12.1	Enniatin A_1_	n.d.	3	0.1	0.1
O-Methylsterigmatocystin	15.0	5	30.3	98.1	Apicidin	n.d.	4	0.3	0.9
Versicolorin C *	10.1	5	29.1	94.9	Beauvericin	77.3	5	197.9	305.6
Versicolorin A *	5.2	5	22.3	36.5	Avenacein Y	n.d.	3	46.2	356.9
Sterigmatocystin	5.0	5	9.3	32.2	Aurofusarin	n.d.	5	22.8	36.2
Mevinolin	22.0	5	22.3	38.3	Fusaproliferin	n.d.	1	n.d.	90.1
Malformin A *	52.8	5	21.6	31.5	Siccanol ^†^	n.d.	4	6994.0	16,420.0
Malformin A_2_ *	0.2	5	0.5	0.8	*Alternaria spp.*				
Secalonic acid D	23.7	5	11.5	37.0	Alternariol	n.d.	5	62.9	207.0
Dechlorogriseofulvin	n.d.	2	n.d.	14.1	Alternariolmethylether	7.0	5	129.4	237.9
Griseofulvin	1.9	5	9.4	37.6	Altertoxi-I	n.d.	5	1.1	7.7
Cycloaspeptide A	n.d.	1	n.d.	37.6	Tentoxin	7.0	5	10.2	23.3
Viridicatin	n.d.	2	n.d.	80.5	Macrosporin	0.8	5	3.8	9.6
Cyclopenol	n.d.	1	n.d.	152.8	*Miscellaneous:*				
Kojic acid	126.0	2	n.d.	56.6	Curvularin	15.2	5	61.9	110.3
Chanoclavin	n.d.	1	n.d.	0.1	Monocerin	16.2	5	191.7	1262.4
*Fusarium spp.*					Emodin	0.9	5	6.4	14.4
Deoxynivalenol	n.d.	3	3.3	235.9	Tryptophol	939.2	5	266.1	503.8
Nivalenol	23.3	4	39.5	15.5	Cytochalasin D	4.9	4	100.8	155.7
Zearalenon	261.0	5	76.7	134.1	Radicicol	n.d.	1	n.d.	3.1
Zearalenon-4-Sulfat	321.8	5	134.1	646.96	Linamarin	n.d.	3	21.4	111.7
α-zearalenol	n.d.	4	1.8	229.92	Lotaustralin	n.d.	3	54.9	320.5
β-zearalenol	3.8	4	2.6	6.2	Orsellinic acid	3249.0	4	1872.0	2264.0
Bikaverin	24.0	2	n.d.	74.0	Brevinamid F	32.6	5	127.2	10.2
Diacetoxyscirpenol	1.2	5	2.2	14.9					

* Semi-quantification based on response of a structurally related compound; ^†^ Number denotes peak area (standard not available); n.d. = less than detection limit, CSC = cottonseed cake, ICT = Islamabad Capital Territory; Control = cottonseed cake collected from a state-owned cattle feed mill in Islamabad Capital Territory.

It should be noted that aflatoxins and *Aspergillus flavus* are already known in the Indian subcontinent to be the common contaminants of cottonseed cake. As early as in 1970’s, 50% samples of cottonseed cake studied in the province of Punjab, Pakistan were found to be contaminated with *A. flavus* which is the predominant fungal spp. producing aflatoxins [5]. In subsequent studies, from 33% to 90% milk samples from various cities of Pakistan were found to be having aflatoxin residues [6,7,8]. This was found to be due to feeding of CSC contaminated with aflatoxin B_1_ [9]. Since last decade, ochratoxins and some of the *Fusarium* trichothecenes have been reported in different feedstuffs in the Indian subcontinent [10,11]. To our knowledge, the tremorgenic mycotoxins being reported by us and andrastin A, equisetin, rubrofusarin, tenuazonic acid, 3-nitropropionic acid have not been previously documented in the subcontinent. Also, present report could be the first documentation of the contamination of CSC with tremorgenic mycotoxins.

### 2.3. Exposure Study

The exposure study was conducted to investigate if the collected CSC samples result in toxicity symptoms under control conditions. The four calves fed the ration based on the control-CSC lost 5.1 kg body weight/head during first 2 weeks of the exposure (Table 3). In the week 3, the weight loss in the two calves on this ration was 3.7 kg/head, while the weight loss for the other two calves fed the CSC-ICT-3 ration in the week 3 was 3.1 kg/head. All the calves were gaining around 2.1 kg body weight per head/week before exposure to the contaminated CSC rations. After the exposure trials, the weight of the animals was monitored for 10 months. These calves gained on an average 1.1 kg per head/week while on the suboptimal grazing conditions at our farm.

The calves fed the control-CSC (also contaminated with mycotoxins) showed labored breathing in the week three. Both of these calves otherwise appeared normal and healthy. On day 21, when these calves were moved to the weighing balance (10 meter distance), one calf fell down and showed symptoms of suffocation. This calf could not stand for 15 min. In our experience, a healthy calf would rise within a couple of seconds, provided it is not exhausted. These symptoms, though noted on only two calves, indicate a possible toxicity of tremorgenic mycotoxins. It may be noted that the levels of the carcinogenic mycotoxins (aflatoxins, ochratoxins) being reported in the present study are exceptionally high. At the levels being reported here, these could alone affect the production and survivability of the dairy animals in a chronic exposure [12]. During an acute exposure of short duration, it appears that the carcinogenic mycotoxins may not be the primary cause of mortality or other health issues. Our deduction form the present investigations is that the buffalo/cattle mortality or toxicity symptoms during acute exposure at dairy farms could be due the contribution of the tremorgenic mycotoxins which have not been considered in the subcontinent previously. The reason is that these mycotoxins were consistently high in all the studied samples. Also, large ruminants are known to withstand aflatoxin levels of up to 0.08 mg/kg body weight (or up to 2500 μg/kg of feed) during acute exposure [13]. The carcinogenic mycotoxins however appear to contribute significantly in the complex symptoms and reduced milk production syndrome noted by the farmers. Previous reports regarding buffalo and cattle mortality due to unknown causes after consumption of CSC in the Cattle Colony of Karachi [2] may also be explained on the basis of these results.

Under the present investigations, the Cry proteins in the CSC samples were lower than the safe range of 10 μg/g. Therefore, it may not be reasonable to allege the GM modification of the cotton for the toxicity and various health issues noted by dairy farmers in recent years. However, it may be possible that the early maturing cotton varieties are more prone to fungal invasion in the climate of the subcontinent. An important factor in this regard is the harvesting season of cotton in South-East Asia. The traditional varieties of cotton used to be harvested in October, whereas the picking up of the cottonseeds from early maturing GM varieties starts from July-August and it is continued through December. This early and long picking season means that the oilseed cake from first picking of seeds is exposed to higher relative humidity during monsoon, *i.e.*, July to mid-September. Also, the seeds during second and third picking could also be exposed to the high relative humidity of the monsoon. This factor alone could significantly contribute in the fungal invasion and mycotoxin development in some batches of the cottonseed cake. Unfortunately, detailed studies on previously grown cotton varieties are not available which limits factual comparison of the early and late maturing cotton varieties under field conditions.

**Table 3:** Results from feeding trials using 35% cottonseed cake rations.

Item	Control ^1^				CSC ICT-3	
	Calf 1	Calf 2	Calf 3	Calf 4	Calf 3	Calf 4
Age (days)	306	344	384	344	-	-
Body weight (kg)	152	152	152	168	-	-
Daily feed intake 1st week (kg)	3.1	3.7	4.0	4.6	-	-
Daily Feed intake 2nd week (kg)	4.3	2.3	4.1	5.1	-	-
Daily Feed intake 3rd week (kg)	3.8	1.2	n.d.	n.d.	3.8	4.2
Weight change (kg)	−7.0	−16.5	−2.0	−2.5	−3.3	−3.0
	(21 days)	(21 days)	(14 days)	(14 days)	(7 days)	(7 days)
Symptoms noted in 3rd week:						
Labored breathing on excitement	+	+	n.d.	n.d.	n.d.	n.d.
Inability to stand ^2^	+	n.d.	n.d.	n.d.	n.d.	n.d.

^1^ The control CSC was also found to be contaminated, though less than the other collected samples, with mycotoxins; ^2^ One calf fell down and showed signs of labored breathing while it was moved towards the weighing scale after 3 week feeding. This calf could not stand for 10 min. All calves otherwise appeared normal while standing and feeding; CSC = cottonseed cake, ICT = Islamabad Capital Territory, + = positive, n.d. = symptoms not apparent; Control = cottonseed cake collected from a state-owned cattle feed mill in Islamabad Capital Territory.

The aforementioned effects of early maturing cotton crop may aggravate further due to nonexistence of established procedures and protocols for cottonseed collection and storage. Traditionally, the labor engaged in cotton collection starts the picking of cotton early in the morning before drying of dew. Though the freshly picked cotton is laid out on ground directly under sun, the hygroscopic nature of the product does not allow it’s drying [14] particularly in the monsoon with a relative humidity of over 80%. The cotton collected from farmers is exposed to harsh weather conditions throughout the value added chain. Even the cotton ginning sector sometimes store cotton on open ground. These processing and storage conditions predispose the cottonseed to fungal invasion and subsequent mycotoxin development, especially in case of unexpected rains. Keeping in view these practices and the specific weather conditions of the country, it is critical to give special attention to the processing of the early maturing cotton varieties. Long term studies are, however, needed in this direction.

## 3. Experimental Section

### 3.1. Collection of Sample and Information

In total five samples of CSC with traceable feeding problems were studied along with a control. Four samples of CSC were collected from CSC retailers in vicinities of Islamabad Capital Territory (ICT) in the year 2011/12. Islamabad is a rain-fed area that is not suitable for oilseed production. The CSC for the dairy farmers of ICT is transported from all cotton production zones of the country. Out of these four samples, one (ICT-1) was suspected to cause mortality at a dairy farm in ICT and this sample originated from district Okara, Punjab. The other three CSC samples (ICT-2, ICT-3, and ICT-4) were suspected to result in miscellaneous health issues at some farms or feed refusal at others. These samples originated from different cities of the provinces of Sindh and Punjab (Shahdadkot, Jhang, and Faisalabad). The fifth CSC sample (CSC-Okara) was submitted to our laboratory in 2013 from a state-owned farm in Okara, Punjab. This sample was known to result in cattle mortality (symptoms not disclosed by the client). The origin of this sample was not known. The control was collected from a state owned cattle feed mill in the ICT. This mill was using CSC at the rate of 5 to 15% in the finished feed, and no feed refusal issues were known against the feed.

The history of toxicity associated with the CSC samples was collected from the farm managers, and retailers. The oil expellers involved in the production of these CSC samples were contacted for getting their perspective and information on complaints regarding CSC feeding problems.

### 3.2. Nutrient, Heavy Metal, and Cry Protein Analyses

The CSC samples were analyzed for proximate and fiber composition, and heavy metal content following protocols specified by AOAC [15]. The equipment used for this purpose are detailed in the supplement. To confirm if the samples were from late maturing cotton varieties, the levels of Cry1Ac proteins in the CSC samples were determined using Quantiplate ELISA kits for Cry1Ab/Cry1Ac (Envirologix, Portland, OR, USA) following protocols specified by the manufacturer. These specific kits were used due to the nature of the late maturing cotton varieties currently being grown in Pakistan.

### 3.3. Fungal and Bacterial Contamination and Their Toxins

The samples were subjected to conventional microbiological procedures (using Oxoid media, Oxoid Ltd., Basingstoke, Hampshire, UK) for assessment of fungal and bacterial contamination [16]. In brief, isolation of Salmonella was performed on MacConkey agar after two stage pre-enrichment of the samples in buffered peptone water and selenite broth. Viable bacteria and coliforms were enumerated on nutrient and MacConkey agar, respectively, using tenfold dilutions in sterile distilled water. For cultivation of fungi, the CSC samples were directly plated simultaneously on Sabouraud agar and filter papers in moist chambers.

The mycotoxins and bacterial toxins were determined at IFA, Tulln using an HPLC-ESI-MS/MS multi-toxin method [17], which has recently been extended to cover around 350 metabolites using a more sensitive mass spectrometer. In brief, 2.5 g ground sample was extracted for 90 min using 20 mL of acetonitrile/water/acetic acid (79:20:1, *v*/*v*/*v*). The raw extract was diluted at the ratio of 1:1 with acetonitrile/water/acetic acid (20:79:1, *v*/*v*/*v*), and directly injected into the LC-ESI-MS/MS system (injection volume 5 μL). Chromatographic separation was performed at 25 °C on a Gemini^®^ C18-column (150 × 4.6 mm i.d., 5 μm particle size) equipped with a C18 4 × 3 mm i.d. security guard cartridge (all from Phenomenex, Torrence, CA, USA), and coupled to a 1290 Series HPLC System (Agilent, Waldbronn, Germany). An LC-MS/MS System (QTrap 5500, Applied Biosystems, Foster City, CA, USA) was used to detect and quantify the bacterial and fungal metabolites. The screening and quantification were performed in the selected reaction monitoring (SRM) mode. Two multiple reaction monitoring (MRM) transitions were acquired per analyte following the Commission decision 2002/657/EC. This yielded 4 identification points for unambiguous identification. In addition, the retention time and the intensity ratio of the two MRM transitions had to agree with the related values of an authentic standard within 2.5% rel. and 30% rel., respectively. External calibration was performed using serial dilutions of a multi-analyte stock solution. The limits of detection and quantification for the fungal metabolites are presented in supplementary Table S3.

### 3.4. Exposure Study

The exposure trial was approved by the Animal Welfare Committee of the National Agriculture Research Centre, Islamabad. The duration of the exposure study was restricted to be only until the appearance of the first signs of any toxicity or up to 21 days of feeding. Another restraint for the exposure study was the availability of the samples in only limited quantities. Therefore, only the CSC-ICT-3 and control were used in the exposure study. Though not known to cause health issues in the finished feed, it should be noted that the control CSC sample was also found to contain a variety of mycotoxins in lab analyses. Therefore, the exposure trial was important to validate the laboratory analyses.

Four buffalo calves (age 10–13 months, body weight 152–168 kg) were allocated to a ration based on the CSC-control for 14 days. Out of these four calves, two were continued on the same ration for another 7 days, while the other two were fed a ration based on the CSC-ICT-3 for 7 days. The ingredient composition (%) of the experimental diets was as follows: Cottonseed cake, 35; wheat bran 10; corn, 10; molasses, 9; wheat straw, 35; and mineral premix, 1. To mask the effect of any bad odors in the cottonseed cake samples, molasses was added in the last step while preparation of the feed. It may be of interest to mention that most farmers in Pakistan use CSC and grain byproducts at a ratio of 1:1 in dairy concentrate supplements. Traditionally, the dairy supplements are mixed with roughages before feeding. The experimental diets used in the exposure study were therefore formulated to mimic the actual CSC feeding scenario in the field.

## 4. Conclusions

Overall, the present study revealed presence of a cocktail of mycotoxins in exceptionally high quantities in the CSC samples suspected to be toxic to cattle. The mycotoxin contamination of the CSC samples was possibly due to substandard processing and storage of the cottonseed and cake.

## Supplementary Materials

Supplementary materials can be accessed at: http://www.mdpi.com/2072-6651/7/6/2188/s1.
