# Supplementary material for: Mycotoxin Cocktail in the Samples of Oilseed Cake from Early Maturing Cotton Varieties Associated with Cattle Feeding Problems
Source: Toxins (Basel). 2015 Jun 12;7(6):2188–97. doi: 10.3390/toxins7062188 (PMC4488697; doi:10.3390/toxins7062188)
Supplement: Supplementary file 1 [file toxins-07-02188-s001.pdf]

Supplementary Information

Nutrient, and Heavy Metal Analyses

The crude protein content of the CSC was estimated using an auto Kjeldahl system (Digest System K-437 and Auto Kjeldahl Unit K-370, Büchi Labortechnik AG, Flawil, Switzerland). Calibrations and nitrogen recovery for protein estimation were done performed using glycine and urea as a standard. The fat and fiber determinations were carried out on a fat extractor (Ankom XT151 Extractor, Macedon, NY, USA) and a fiber analyzer (Ankom A2000I Fiber Analyzer, Ankom, Macedon, NY, USA), respectively using standardized protocols. Calibrations were performed using samples known for their fat and fiber contents. For determination of heavy metals, the samples were digested using nitric and hydrochloric acids and then analyzed for the levels of lead (Pb), chromium (Cr), cadmium (Cd), copper (Cu), and nickel (Ni) using an atomic absorption spectrometer (AAnalyst 700, Perkin Elmer, Waltham, MA, USA).

Table S1. Nutrient analyses (as such basis) of the cottonseed cake samples.

Item	Mortality samples		Feed refusal samples			Control
	CSC Okara	CSC ICT-1	CSC ICT-2	CSC ICT-3	CSC ICT-4	
Collection date	January 2013	April 2012	November 2011	February 2012	April 2012	February 2012
Nutrients (g/100 g):						
Dry mater	90.9	90.9	92.4	92.4	92.5	92.8
Crude protein	23.1	25.4	26.1	24.3	28.2	21.9
Ether extract	7.3	6.7	6.8	6.8	6.6	9.1
Ash	4.5	5.0	4.9	5.0	3.8	4.5
Crude fiber	29.3	27.3	28.9	29.3	28.9	26.3
NDF	65.5	53.6	57.5	60.1	57.2	50.2
ADF	43.6	39.7	45.5	49.1	44.9	37.9
ADL	12.5	11.1	10.3	10.8	14.1	8.4

NDF = neutral detergent fiber, ADF = acid detergent fiber, ADL = acid detergent lignin, ICT = Islamabad Capital Territory. Control = cottonseed cake collected from a state-owned cattle feed mill in Islamabad Capital Territory.

Table S2. Heavy metal content, and bacterial counts in the cottonseed cake samples.

Item	Mortality samples		Feed refusal samples			Control
	CSC Okara	CSC ICT-1	CSC ICT-2	CSC ICT-3	CSC ICT-4	
Heavy metals (mg/kg):						
Lead	5.8	10.3	3.0	n.d.	7.0	n.d.
Chromium	3.2	n.d.	4.0	n.d.	n.d.	n.d.
Cadmium	3.1	1.1	1.9	0.6	3.1	n.d.
Bacteriological examination:						
Total counts	3.9×10^6	2.0×10^3	8.0×10^5	4.4×10^6	8.2×10^7	1.1×10^7
Coliform	+	+	+	+	+	+
Salmonella	n.d.	n.d.	n.d.	n.d.	n.d.	n.d.

n.d. = less than detection limit or negative, ICT = Islamabad Capital Territory, + = positive. Control = cottonseed cake collected from a state-owned cattle feed mill in Islamabad Capital Territory. Maximum tolerable limit in feeds (mg/kg): lead = 100; chromium = 100; cadmium = 1.

Table S3. Limits of Detection and Quantification of the positively identified fungal metabolites in the cottonseed cake samples (µg/kg).

Metabolite	LOD	LOQ	Metabolite	LOD	LOQ
Aflatoxin B_1	0.3	1	Deoxynivalenol	1	3
Aflatoxin B_2	0.5	1.5	Nivalenol	0.8	2.5
Aflatrem †	-	-	Zearalenon	0.3	1
Cyclopiazonic acid	20	60	Zearalenon-4-Sulfat	0.4	1.2
Ochratoxin A	0.3	1	α-zearalenol	0.5	1.5
Ochratoxin B	0.5	1.5	β-zearalenol	0.5	1.5
Citrinin	20	60	Bikaverin	4	12
Andrastin A †	-	-	Diacetoxyscirpenol	0.15	0.5
Paspalitrem A †	-	-	Monoactoxyscirpenol	1	3
Paspalin †	-	-	Moniliformin	0.5	1.5
Paspalinin †	-	-	Enniatin B	0.01	0.03
Equisetin	0.05	0.15	Enniatin B_1	0.02	0.06
Rubrofusarin	-	-	Enniatin A_1	0.02	0.06
Tenuazonic acid	3	10	Apicidin	0.1	0.3
3-Nitropropionic acid	0.8	2.5	Beauvericin	0.04	0.12
Averufin	0.04	0.15	Avenacein Y	5	15
Averufanin *	0.04	0.15	Aurofusarin	4	12
Nidurufin *	0.04	0.15	Fusaproliferin	15	45
Averantin	0.04	0.15	Alternariol	0.5	1.5
Norsolorinic acid *	0.04	0.15	Alternariolmethylether	0.05	0.15
O-Methylsterigmatocystin	0.25	0.8	Altertoxin-I	0.3	1
Versicolorin C *	0.04	0.15	Tentoxin	0.2	0.6
Versicolorin A *	0.04	0.15	Curvularin	0.4	1.2
Sterigmatocystin	0.1	0.3	Siccanol †	-	-
Mevinolin	2	6	Monocerin	0.4	1.2
Malformin A *	0.05	0.15	Physcion	5	15
Malformin C	0.05	0.15	Macrosporin	0.2	0.6
Malformin A_2 *	0.05	0.15	Emodin	0.08	0.25
Secalonic acid D	2.5	7.5	Chanoclavin	0.01	0.03
Dechlorogriseofulvin	0.5	1.5	Tryptophol	15	45
Griseofulvin	0.5	1.5	Cytochalasin D	0.2	0.6
Cycloaspeptide A	1	3	Radicicol	1	3
Viridicatin	0.3	1	Linamarin	2.5	7.5
O-Methylviridicatin	0.3	1	Lotaustralin	0.8	2.5
Cyclopenol	0.8	2.5	Orsellinic acid	50	150
Kojic acid	15	45	Brevianamid F	0.4	1.2

LOD = Limit of Detection, LOQ = Limit of Quantification, * Semi-quantification based on response of a structurally related compound; † Number denotes peak area (standard not available).
